# I'd Rather Be . . . : Activities Older Adults Reported Leading to Decreased Engagement With Cognitive Training

**DOI:** 10.1093/geroni/igaa057.1042

**Published:** 2020-12-16

**Authors:** Erin Harrell, Nelson Roque

**Affiliations:** 1 University of Alabama, Tuscaloosa, Tuscaloosa, Alabama, United States; 2 Pennsylvania State University, University Park, Pennsylvania, United States

## Abstract

One modifiable risk factor of dementia is cognitive inactivity. Given cognitive ability is closely tied to continual performance of instrumental activities of daily living, cognitive training programs continue to be explored as a way to boost cognition and allow older adults to remain independent longer. While the efficacy of cognitive training is controversial, identifying activities older adults are willing to limit in exchange for cognitive training provides valuable information in relation to designing cognitive training programs that appeal to older adults. Using a qualitative approach, this study highlights activities older adults (ages 64+) noted as contributing to decreased gameplay of a cognitive training program on a tablet device. We found that respondents (61%) noted playing less as a result of entertainment activities (i.e., reading and playing games), social activities (31%) and travel (27%). Findings have implications for device form factor in administering cognitive training and other programs.

